# Energy security: the role of shale technology

**DOI:** 10.1007/s11356-023-25654-w

**Published:** 2023-02-09

**Authors:** Masoud Shirazi

**Affiliations:** 1 CeBER and Faculty of Economics, University of Coimbra, Coimbra, Portugal; 2 Cyprus Institute of Marketing, Nicosia, Cyprus

**Keywords:** Energy security, Portfolio decision, Shale technology, Asymmetric behavior, Markov Switching Model, Q47, Q42, Q37

## Abstract

Sustainable energy systems are sensitive to the countries’ energy portfolio decisions, shaping geopolitics and contributing to the global energy security (ES). Accordingly, this paper applies the “Markov regime-switching” method to explore the impact of “the North American shale technology” (NAST) on behavioral regimes of the US energy security measurements (ESM), e.g., diversity of primary energy demand (${\mathrm{ESI}}_{\mathrm{I}}$), net energy import dependence (${\mathrm{ESI}}_{\mathrm{II}}$), non-fossil fuel resource portfolio (${\mathrm{ESI}}_{\mathrm{III}}$), and crude oil import dependency (${\mathrm{ESI}}_{\mathrm{IV}}$). The findings confirm time-varying and asymmetric behavior of the US ESM before and after the NAST. Specifically, the overall interaction of substitution effect and scale effect of NAST strengthens the US energy systems through ${\mathrm{ESI}}_{\mathrm{I}}$, ${\mathrm{ESI}}_{\mathrm{III}}$, and ${\mathrm{ESI}}_{\mathrm{IV}}$, while ${\mathrm{ESI}}_{\mathrm{II}}$ leads to higher risks of the US energy supply security. Consequently, the shale reserves development, diversification of primary energy demand and import supply, and advanced energy transport and trading policies, are suggested to overcome the barriers in achieving (i) availability, (ii) accessibility, (iii) affordability, and (iv) acceptability aspects of ES and vulnerability reduction of the US energy systems in respect of risk and resilience.

## Introduction and contribution

### Background

Indeed, energy and the relevant policies are still assessed today as the top challenges ahead to the nation’s future welfare, way of life, and national security. The development of energy systems, i.e., technological dynamics and social complexity, needs to focus on (i) energy equity, (ii) energy security, and (iii) environmental sustainability, called the “energy trilemma” (Bale et al. 2015). Currently and based on Bale et al. (2015), the world’s energy systems are trapped in a carbon-based fuel portfolio ($\mathrm{CFP}$), which is a motivation for energy security (ES) development (Costello 2007; Shahzad 2020). Therefore, this paper aims to analyze dynamic behavioral features of the US ES that relate to vulnerability reduction of the energy systems in terms of risk and resilience.

The issue of ES refers to a wide range of aspects (Yergin 2006), from the classic concept, i.e., affordable and reliable flow of resource supply (Yergin 1998; Colglazier and Deese 1983) to contemporary definitions, e.g., environmental acceptability and accessibility, of energy sources in an economy (Goldthau 2011)^1^. Specifically, ES covers 4 As, including transportation, transmission, and geopolitical accessibility^2^, environmental, political, and social acceptability, immediate physical availability, and price affordability of primary energy sources (Sutrisno et al. 2021).

Particularly, in respect of physical availability, a resource is available when it is plenty enough for keeping on an important recoverable energy source. The economic aspect of ES is described by the price affordability of the resource acquisition. The accessibility feature of ES relates to transmission and transportation barriers, e.g., “long-term sales contracts”, large infrastructure investments, and geopolitical factors, among others. From the viewpoint of environmental acceptability, the issue of ES indicates an economy’s success in switching from fossil fuels and nuclear energy to a new and renewable energy portfolio that lowers environmental degradation. In respect of infrastructure within the country, actions held by developed and developing economies in response to the acceptability concerns are dissimilar. The policies related to environmental, political, and social acceptability for developed countries are focused mainly on how the market mechanism allocates resources. The objective of these countries is to invest in the research and development projects of new and renewable energy sources to capture long-term economic opportunities in their energy systems since major financial constraints are not issued in these economies. For developing countries, acceptability policies are founded on the requirements for renewable energy development, regional cooperation for resources, foreign infrastructure investment, and risk and capital sharing (APERC 2007)^3^. It is likely that new and renewable energy sources not only impact geopolitics but threat and realization of unfavorable geopolitical events, particularly in institutionally and risky unstable situations, can also affect investment decisions in such energy sources by raising the capital cost. These geopolitical acts transfer negative shocks to the energy markets through the asset pricing mechanisms and return channels as the escalation of the regional and international geopolitical tensions adversely influences the energy finance and subsequently ES (Flouros et al. 2022; Øverland et al. 2017).

Hence, policymakers in both energy-exporting and energy-importing countries need to adopt comprehensive dynamic energy policies and therefore, enhance their ESs (Chalvatzis and Ioannidis 2017; Vivoda 2014; Cohen et al. 2011). However, the role of ES on resource- and non-resource sectors, capital formation, technology improvements, and economic growth of the energy-exporting countries is inevitable since they are vulnerable to external market shocks (Nepal and Paija 2019; Griffiths 2017; Bilgili et al. 2016). On the other hand, as an economy is dependent on the imported-primary energy sources to cover its primary energy demand ($\mathrm{PED}$), there is a limited possibility to meet its energy consumption through domestic supply sources, which leads to higher risks and less resilience (capability to respond to the disruptions) of the country’s energy supply security^4^.

Since the 1970s, the ES has been made a priority by Republican and Democratic presidential authorities and policymakers. Yet, a regular tool is still missed to measure the nation’s improvement and then assess the effect of policies on the United States (US) ES. Compared with 1980, the USA was one of 15 countries with a 2018 risk score^5^ lower than its initial 1980 score, from 1071 (its highest risk score in the record) to 727, a drop of nearly one-third. The second and third world’s lowest ES risk scores are established for New Zealand and Canada with 757 and 802 scores, respectively. Accordingly, for the USA with the world’s lowest ES risk score, the first energy-usage rank of such economy among 25 large energy-consuming countries intensifies the importance of monitoring the time-varying behavioral characteristics of ES that is necessary to develop the 4 As dimensions of ES and hence, remain less vulnerable in terms of risk and resilience, in response to the market shocks of energy resources (Global Energy Institute. The US Chamber of Commerce 2020)^6^.

The US crude oil and natural gas production, particularly from primarily deep shales (geological or tight oil formations) have been increased through merging the “horizontal drilling” with “hydraulic fracturing” technologies, called the “shale technology”^7^. The focus of the US shale production has been shifted from volumes to efficiency and overall performance rates improvement. As a result, the industry has switched to focus considerably on infrastructure, logistics, and the supply chain optimization (Scholl 2019). However, investment in oil and gas infrastructure is rarely a plain affair. Due to remarkable uncertainties in future energy prices, geopolitical and regulatory challenges, and the large scales of investments, projects often meet cost overruns and schedule delays. In particular cases, the interests behind the investment plans might have to be shifted away (Tan and Barton 2017). Companies intend to extend the applied policies that caused bumper profits in 2021, and shale activists are cautious of an investor's retaliation if they increase spending too rapidly. But the issue of capital discipline is developing. It is perhaps necessary and safer for the companies to expand again, though at slower rates. Underpinning the profitability, however, is unsustainable levels of investment. In 2021, most US shale companies reinvested smaller than 50% of their cash flows in new drilling activities, as the industry shifted downward into the “maintenance capex” regime. But initial wells production rates usually decrease quickly, so the companies require to drill continuously for sustainable output. Hence, they can just pull back on suggested investment continually without sacrificing future levels of production and cash generation (Cahill 2022). In respect of the outcomes, the “shale technology” decreases the natural gas cost of production by declining the ${\mathrm{CO}}_{2}$ separation costs via potential technical and economic infrastructure, lowering the natural gas price. Also, the intermediate technology of the shale gas mitigates the US short-term environmental concerns since the reduced prices of natural gas can decrease the energy trilemma concerns (Acemoglu et al. 2019)^8^. Accordingly, the “North-American shale technology” (NAST) is considered a potential determining factor to analyze the short- and long-term behavioral properties of the US energy systems.

### Contribution of the study

This article aims to fill in the knowledge gap found throughout the literature in the field of ES as follows:

First, and based on (APERC 2007) classifications, the actual time-series of four behavioral indices, e.g., “diversification of primary energy demand” ($\mathrm{DoPED}$), “net energy import dependency” ($\mathrm{NEID}$), “non-carbon-based fuel portfolio” ($\mathrm{NCFP}$), and “net oil import dependency” ($\mathrm{NOID}$), are calculated for the US economy during the period January 1973–April 2021, to analyze the behavior of the US ES before- and after the NAST. To this end, the suggested time period is divided, using the breakpoint in year 2006 as the outset of the NAST (Shirazi and Šimurina 2022; Shirazi et al. 2021; Geng et al. 2016)^9^.

Second, the time-series of the long-term trends and short-term fluctuations of the actual ESM are extracted, using the Hodrick and Prescott (1997) filter suggested by Ewing and Thompson (2007). This decomposition helps to recognize the impact of NAST on the long-term trends as well as the magnitude, time duration, and the number of cyclical movements (ups and downs) of the mentioned indices to follow the behavioral characteristics, e.g., risk and resilience, of the US ES.

Finally, the interconnection of uncertainty, speed- and expected duration of the specified states through the “Markov switching autoregressive method with regime heteroskedasticity” ($\mathrm{MSARH}$) is focused to explore the potential asymmetric and time-varying behavioral switching regimes of the US’ ESM, in response to the NAST (Shirazi and Šimurina 2022; Shirazi et al. 2021; Geng et al. 2016)^10^.

Consequently, the comparative analysis of the findings leads to identifying the US “portfolio decisions of primary energy sources” ($\mathrm{PDPES}$), declining risks and promote resilience of energy systems, i.e., the equitability, diversification and imports, and ${\mathrm{CO}}_{2}$-related environmental degradation, by figuring out its main strengths and weaknesses^11^.

Therefore, to understand the impact of the NAST on the behavioral characteristics regarding the performance of the US ES, the following research questions are investigated:

- What is the difference in the behavior of actual, long-term trends, and short-term fluctuations of the US ESM, e.g., ${\mathrm{ESI}}_{\mathrm{I}}$, ${\mathrm{ESI}}_{\mathrm{II}}$, ${\mathrm{ESI}}_{\mathrm{III}}$, and ${\mathrm{ESI}}_{\mathrm{IV}}$, pre-and post (p&p)-the NAST?
- How are the behavioral features of the switching regimes (e.g., typical state, uncertainty, and speed of the regimes) of the US ESM explained p&p-the NAST?
- How is the US ES affected by the interconnection of uncertainty, speed- and expected duration of specified switching regimes of the measurements in response to the NAST?

The overall findings of this paper support the time-varying and asymmetric behavior of the US ESM p&p-the NAST. Specifically, the equitability dimension of the US ES are developed by the NAST that leads to a combination of fewer risks and higher resilience of the US energy supply security. Also, a mixture of higher risk and less resilience is found for the US energy supply security after the NAST, because the country has been getting highly relies on energy imports and therefore, there is a limited possibility to meet its energy consumption through domestic supply sources. Moreover, results imply that the NAST improves the contribution level of hydro, nuclear, and new and renewable energy sources ($\mathrm{NRE}$) to total $\mathrm{PED}$ in the US primary energy system, and hence, a considerable decline of the US' ${\mathrm{CO}}_{2}$-related environmental degradation is concluded^12^.

## Literature and theory

The first classification of recent studies regarding availability and accessibility dimensions of ES focuses on the impact of energy sources’ regional and international trade networks on ES (Tuchinda et al. 2021; Peng et al. 2021; Shirazi et al. 2021; Shepard and Pratson 2020; Dong et al. 2020; Rodríguez-Fernandez et al. 2020; Shirazi et al. 2020; 2019; Maltby 2013) and concludes that ES significantly depends on reliable trade relationships throughout global trade networks of both renewables and non-renewables.

The second group of articles investigates determining the risks around ES, e.g., environment, technology, energy supply, geopolitics, and economic factors of individual economies and regions (Kosai and Unesaki 2020a; García Mazo et al. 2020; Hasanov et al. 2020; Karatayev and Hall 2020; Lin and Raza 2020; San-Akca et al. 2020; Liu et al. 2020a, b; Sun et al. 2020; Groissböck and Gusmão 2020; Zeng et al. 2017; Kiriyama and Kajikawa 2014; Francés et al. 2013; Roques et al. 2008) and finds that $\mathrm{DoPED}$, renewables development, citizen commitment, the mobilization of technological and economic resources, and finally, a model of generation, efficiency, and distribution as well as the preventive- and optimizing control models have constructive roles in optimization of the security status and therefore, ES enhancement.

The third category of literature analyzes the performance of ES level based on indicators (Shirazi and Fuinhas 2023; Gong et al. 2021; Li et al. 2020; Augutis et al. 2020; Kosai and Unesaki 2020b; Gasser 2020; Yuan and Lu 2019; Sarangi et al. 2019; Li and Chang 2019; Le and Nguyen 2019; Gan et al. 2019; Wang and Zhou 2017; Kosai and Unesaki 2017; García-Gusano et al. 2017; Anvar 2016; Kisel et al. 2016; Ang et al. 2015; Thangavelu et al. 2015; Martchamadol and Kumar 2014; 2013; Gracceva and Zeniewski 2013; Wu et al. 2012; Augutis et al. 2012; Stirling 2010; Kruyt et al. 2009; Scheepers et al. 2006) and exhibits that strategic management, storage and control of resource supply, higher reserves of energy sources, clean energy development, optimization of the energy-consuming terminal structures, energy efficiency improvement and policy monitoring increase the ES level in the countries under consideration.

The fourth sort of articles considers the use of potential opportunities to improve ESM (Yong et al. 2021; Jiang et al. 2021; Bilgili et al. 2020; Rajavuori and Huhta 2020; Bekhrad et al. 2020; Coester et al. 2020; 2018; Azzuni and Breyer 2018) and illustrates the positive impact of investment screening projects such as integrated energy systems on ES enhancement that is applicable through wave energy, cross-country transactions in resource infrastructures, energy hub security region, subsidized investing in renewable energy technologies, e.g., storage and controlling technologies, data-intensive energy technologies including the digitalization process of the energy systems, and the shale development.

Also, from the view of the energy dilemma, the comparative analysis between the transition towards renewable energy sources and prioritizing fossil fuels as reliable supplies is investigated (Taherahmadi et al. 2021; Mabea 2020; Pérez et al. 2019; Novikau 2019; Gillessen et al. 2019; Lu et al. 2019; Zaman and Brudermann 2018; Jun et al. 2009). They conclude that focusing on renewables lowers the import dependence of the economy, while reliable supplies through transmission and storage capability can mitigate the volatility and costs of the energy environment. Also, the combination of ES perspectives and energy governance helps developing countries to prevail the barriers of the energy transition process.

Finally, some recent articles investigate the impact of oil price shocks (Babajide 2017; Peersman and Van Robays 2012; Van Hove 1993) and energy intensity (Tvaronavičienė 2016; Tvaronavičienė et al. 2015; Dezellus et al. 2015; Dzemyda and Raudeliūnienė 2014; Raudeliūnienė et al. 2014) on the energy market. The most related conclusion to ESM that the oil shocks lead to breaks in consumption patterns. Also, they show that the development of sustainable entrepreneurship and energy stewardship has a positive impact on ES.

Therefore, the studies above, however, show no implications for the nexus between the “shale technology” and the behavioral features of the ESM, specifically for the US economy as the biggest world’s energy user (APERC 2007). Especially, the US ES is affected by the NAST, through the substitution effect and scale effect (Acemoglu et al. 2019; Kuuskraa et al. 2013). Based on the substitution effect, the process of the NAST facilitates the substitution of coal, oil, and green energy sources (e.g., nuclear and renewables) by natural gas throughout the energy portfolio that can enhance $\mathrm{DoPED}$. Moreover, the high-carbon replacement effect (coal- and crude oil replacement via natural gas) reduces the country's ${\mathrm{CO}}_{2}$ emissions. By contrast, the low-carbon energy-related substitution effect (natural gas-clean energy sources replacement effect) causes higher ${\mathrm{CO}}_{2}$ emissions. It is generally supposed that the overall substitution effect can potentially decrease ${\mathrm{CO}}_{2}$ emissions from resource consumption since the high-carbon replacement effect dominates the low-carbon substitution effect and hence, promotes the $\mathrm{NCFP}$, i.e., low ${\mathrm{CO}}_{2}$-related environmental degradation, of the economy. Besides, the NAST through the scale effect contributes to a price reduction of the energy sources, supposed to have negative effects on the US NOID as well as $\mathrm{NEID}$, which causes the US ES enhancement through the possibility to meet its energy consumption via domestic supply sources^13^.

Accordingly, the efficient $\mathrm{DoPED}$ should be utilized to cause the US long-term ES. The US ES is analyzed on this paper through the 4 As dimensions of primary energy resources, e.g., coal, crude oil, natural gas, hydroelectric power, and $\mathrm{NRE}$. To this end, four indices, e.g., $\mathrm{DoPED}$, $\mathrm{NEID}$, NCFP, and NCFP are calculated to expose the importance and potential risks and benefits, regarding the US' $\mathrm{PDPES}$ p&p-the NAST (APERC 2007). Then, the applicable and comprehensive energy policies are suggested as important factors affecting the structure of energy conservation and vulnerability reduction, i.e., low risk and high resilience, to increase ES and promote sustainable economic development.

- *DoPED: ESI*_*I*_
  $\mathrm{DoPED}$ balances the energy mix to cope with the market shocks of energy resources that lead to volatility reduction of fuel prices, contributes to energy price stability, and promotes the availability, affordability, and accessibility aspects of ES, based on the preferred objective priorities of the energy systems (Francés et al. 2013). The Shannon index is modified to develop $\mathrm{DoPED}$ and measure biodiversity, which is presented by ES indicator $\mathrm{I}$ (${\mathrm{ESI}}_{\mathrm{I}}$). Therefore, ${\mathrm{ESI}}_{\mathrm{I}}$ exhibits the equitability dimension of the US $\mathrm{DoPED}$ that is shown below:
  1 $$\mathrm{D}= -\sum\nolimits_{\mathrm{i}=1}^{\mathrm{T}}({\mathrm{P}}_{\mathrm{i}}{\mathrm{lnP}}_{\mathrm{i}})$$
  2 $${\mathrm{ESI}}_{\mathrm{I}}=\mathrm{DoPED}= \frac{\mathrm{D}}{{\mathrm{D}}_{\mathrm{max}}}\times 100$$

where $\mathrm{D}$ is Shannon’s diversity index, ${\mathrm{P}}_{\mathrm{i}}$ shows the share of primary energy source $\mathrm{i}$ in total $\mathrm{PED}$, ${\mathrm{D}}_{\mathrm{max}}$ displays the maximum value of $\mathrm{D}$, and $\mathrm{i}=(1, 2, \dots ,\mathrm{ T})$ is used to indicate $\mathrm{T}$ types of primary energy sources. As the indicator is calculated close to zero, the country is dependent on one primary energy source, while a value close to 100 indicates that the economy’s energy supply sources are equally distributed among the major primary energy sources. Thus, a fewer risk of the US ES is concluded as a higher indicator’s value is assessed. The graphical results of the Hodrick and Prescott (1997) filter for ${\mathrm{ESI}}_{\mathrm{I}}$ are shown in Fig. 3.

b. *NEID:* ESI_II_

The second ES indicator is the US $\mathrm{NEID}$. The Shannon index is also transformed to measure the effect of diversification and imports on ES. The second indicator (${\mathrm{ESI}}_{\mathrm{II}}$) for the US economy is weighted by the energy consumption intensity of each primary energy source as follows:

3 $$\mathrm{D}= -\sum\nolimits_{\mathrm{i}=1}^{\mathrm{T}}({\mathrm{C}}_{\mathrm{i}}{\mathrm{P}}_{\mathrm{i}}{\mathrm{lnP}}_{\mathrm{i}})$$

$$\mathrm{s}.\mathrm{t}: {\mathrm{C}}_{\mathrm{i}}=1- {\mathrm{m}}_{\mathrm{i}}$$

4 $${\mathrm{DoPED}}_{\mathrm{Import}\;\mathrm{Reflective}}=\frac{\mathrm D}{{\mathrm D}_{\max}}$$

5 $${\mathrm{ESI}}_{\mathrm{II}}=\mathrm{NEID}=1-\frac{{\mathrm{DoPED}}_{\mathrm{Import}\;\mathrm{Reflective}}}{{\mathrm{ESI}}_{\mathrm I}}$$

where ${\mathrm{C}}_{\mathrm{i}}$ correction factor for ${\mathrm{P}}_{\mathrm{i}}$, ${\mathrm{D}}_{\mathrm{max}}$ the maximum value of $\mathrm{D}$, and ${\mathrm{m}}_{\mathrm{i}}$ is used to indicate the share of net primary energy import in energy source $\mathrm{i}$. So, the US economy is dependent on domestic primary energy sources to cover its $\mathrm{PED}$ as the final value is closer to zero. Conversely, a value close to 100% exhibits that the country highly relies on energy imports and there is a limited possibility to meet its energy consumption through domestic supply sources. Hence, a higher risk of ES is concluded as a higher indicator’s value is determined. The graphs of the actual, the cycle, and the trend calculations of ESI_II_ are depicted in Fig. 4.

iii. *NCFP:* ESI_III_

The third ES indicator (${\mathrm{ESI}}_{\mathrm{III}}$) reflexes the US’ economy’s success to switch from a $\mathrm{CFP}$ to $\mathrm{NCFP}$. The third indicator implies the contribution level of hydro, nuclear, and $\mathrm{NRE}$ to total $\mathrm{PED}$, shown as follows:

6 $${\mathrm{ESI}}_{\mathrm{III}}=\mathrm{NCFP}=\frac{\mathrm{Hydro}\;\mathrm{PED}+\mathrm{Nuclear}\;\mathrm{PED}+\mathrm{NRE}\;\mathrm{PED}}{\mathrm{Total}\;\mathrm{PED}}$$

The $\mathrm{NCFP}$ indicator quantifies the progress of a country’s diversification towards alternative energy sources by improving the share of non-fossil fuel energy sources (nuclear, and new and renewable energies) applied to meet energy consumption. Therefore, a considerable potential offset to lower ${\mathrm{CO}}_{2}$-related environmental degradation of the US ES is concluded as a higher indicator’s value is calculated. The graphical presentation of the calculated ${\mathrm{ESI}}_{\mathrm{III}}$, and its short-term fluctuations, and long-term trend are depicted in Fig. 5.

iv. NOID: ESI_IV_

The share of the US economy’s net oil imports in its total $\mathrm{PED}$ is utilized as the fourth ES indicator to calculate the country’s $\mathrm{NOID}$. The suggested indicator is presented below:

7 $${\mathrm{ESI}}_{\mathrm{IV}}=\mathrm{NOID}=\frac{\mathrm{Net}\;\mathrm{Crude}\;\mathrm{Oil}\;\mathrm{Imports}}{\mathrm{Total}\;\mathrm{PED}}$$

Consequently, a higher risk of the US ES is determined as a higher indicator’s value is measured. The calculated time series of the actual ${\mathrm{ESI}}_{\mathrm{IV}}$ for the US economy and its decomposition into cyclical movements and long-term trend are presented in Fig. 6.

## Material and methods

### Material

In order to calculate the actual time-series of the US ESM, the consumption and net import data in billion cubic feet for each primary energy source, e.g., coal, natural gas, crude oil, hydro-electric power, nuclear and new and renewable energy are collected from the US Energy Information Administration (EIA)/Monthly Energy Review, August 2021 for the period January 1973–April 2021. Also, the impact of NAST on the behavioral characteristics of the US ESM are examined through divided time periods, using the breakpoint in year 2006 as the beginning of the NAST (Shirazi and Šimurina 2022; Shirazi et al. 2021; Geng et al. 2016). Specifically, the US primary energy market is found to have overlapped with numerous structural break points, during the period of the global financial crisis. Therefore, the role of the financial crisis mentioned above is eliminated to meet the specific effects of NAST on ESM of the US economy without bias. Consequently, the period of time during the beginning of 1973, January to the first of January 2006 is suggested as pre-the NAST, and the time period between 1 of September 2009 and the end of April 2021 is considered as post-the NAST (Shirazi and Šimurina 2022; Shirazi et al. 2021; Geng et al. 2016; Aruga 2016)^14^.

### Methods

#### The HP filter

**Fig. 1:**
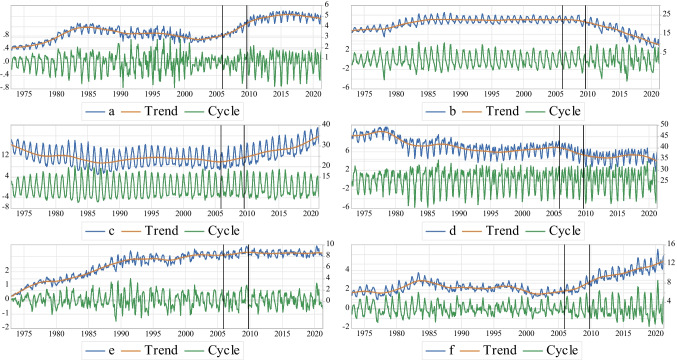
The share of each $\mathrm{PED}$ to total $\mathrm{PED}$;** a** Biomass, **b** Coal, **c** Natural Gas, **d** Petroleum, **e** Nuclear, **f** Total Renewable

In order to find any potential changes experienced by each source of primary energy, e.g., the renewable and non-renewable resources of the US energy system during the time period under consideration, the Hodrick and Prescott (1997) filter is applied in this paper to decompose the actual time-series of primary energy sources to the cyclical movements (short-term fluctuations) and long-term trend of the US economy p&p-the NAST^15^. Based on Fig. 1, the calculated share of each $\mathrm{PED}$, e.g., biomass (a), coal (b), natural gas (c), petroleum (d), nuclear (e), and total renewable (f), to total primary energy consumption ($\mathrm{PED}$) shows an increasing trend after the NAST for biomass (a), natural gas (c) and total renewable (f) resources, while the results indicate a decreasing trend for coal (b) and petroleum (d) with no significant change for nuclear electric power (d). Also, the NAST leads to more short-term fluctuations of biomass (a), coal (b), and total renewable (f), whereas the cyclical movements of natural gas (c), petroleum (d), and nuclear electric power (e) are not significantly affected by the NAST.

**Fig. 2:**
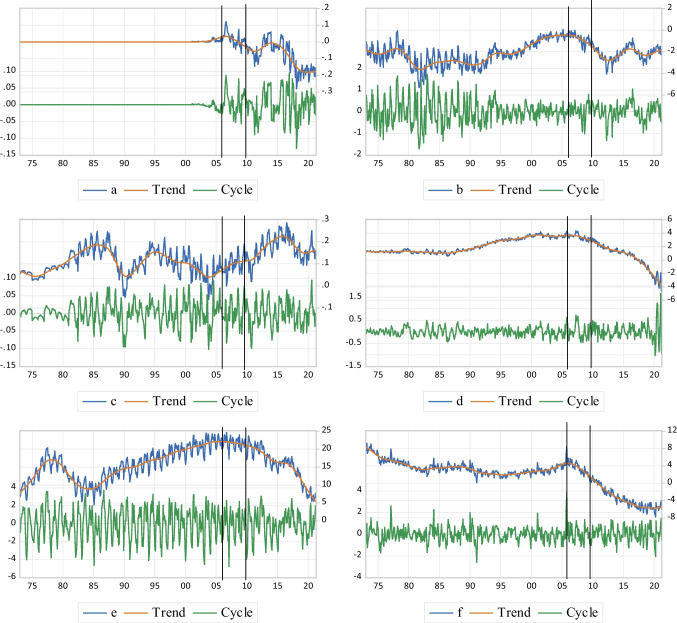
The share of each $\mathrm{PENI}$ to total $\mathrm{PED}$;** a** Biomass, **b** Coal, **c** Electricity, **d** Natural Gas, **e** Crude Oil, **f** Petroleum

Moreover, the findings exhibit a decreasing trend for the share of biomass (a), natural gas (d), crude oil (e), and petroleum (f) net import ($\mathrm{PENI}$) to the total $\mathrm{PED}$ of the US' economy after the NAST, while an increasing trend is detected for electricity (c) as well as clustering ups and downs for the share of coal (b) net import to total $\mathrm{PED}$, following the NAST (Fig. 2). From the other aspect, the short-term fluctuations of the share of biomass (a), coal (b), natural gas (d), and petroleum (f) net import to total $\mathrm{PED}$ are intensified after the NAST, whereas the results show no specific changes for cyclical movements of electricity (c), and crude oil (e) primary energy sources.

Accordingly, the potential impacts of the NAST on the behavioral characteristics of the major ESM should be analyzed, since the US ES depends on the modes and specifications of any changes experienced by each source of primary energy.

#### MSARH

Following Bai and Lam (2019), linear and static regressions are not appropriate for modeling the behavioral regimes of the US ESM, if the characteristics of kurtosis and skewness are determined in the distribution functions of the measurements. The Markov switching technic, introduced by Hamilton (1996), helps to indicate that ESM under different regimes have different characteristics, which are often experienced in the model’s estimates. In this regard, $\mathrm{MSARH}$ can effectively obtain variables’ dynamic characteristics and nonlinearity, which the linear and static regressions do not capture. Therefore, this technic facilitates the change in the ESM to switch between different states, considering any changes over the mentioned time periods. Also, the model explores the regimes of the ESM p&p-the NAST and then reveals whether the NAST has led to the change of the US’ ESM, following their dominant state differences. Accordingly, the behavioral properties of a variable through a nonlinear relation, are assumed for modeling, based on the variation in different regimes. The quantitatively nonlinear models are categorized into two main classifications in respect of the switching speed across the determined regimes. In the first category of the nonlinear models, e.g., “artificial neural networks” and “smooth transition autoregressive (STAR)”, the movement from a specified state to another is determined slowly and moderately. While the regime transition takes place sharply in the second category, e.g., “the Markov regime-switching models ($\mathrm{MRSM}$)” and “Copula method”. The modulation processing depends on the system situation in the STAR and “artificial neural network” models, and therefore, the gradual state-switching process has been assessed. By contrast, the state-change is introduced as an exogenous switching process in the $\mathrm{MRSM}$ (Shirazi and Šimurina 2022; Shirazi et al. 2021). Moreover, the “dynamic conditional method of copula-GARCH” is a flexible technique, which is used to analyze multivariate distributions by modeling heavy tail, volatility clustering, asymmetric relationships, and time-varying correlations, especially through the financial time-series analysis (Bai and Lam 2019; Silva Filho et al. 2014). Notably, the characteristics of peak and thick tails are better explained by $\mathrm{MSARH}$. Despite the number of switching states being pre-identified, the empirical studies suggest that $\mathrm{MSARH}$ models can dominate various drawbacks (Liang et al. 2019; Cheng et al. 2018). First, $\mathrm{MSARH}$ models are able to control multiple equilibria and nonlinearities related to the interaction effects. Second, various time-series characteristics of variables, including non-normality, fat-tail, heteroscedasticity, and time-varying issues are considered. Then, economic cycles are determined endogenously by $\mathrm{MSARH}$ models; hence, it is not required to separate the applied time-series into high and low fluctuations. Lastly, the *p*-values of different states can be explicitly assessed by $\mathrm{MSARH}$ models, particularly the transition probability among switching duration and several economic cycles. Consequently, $\mathrm{MSARH}$ relates to the theoretical hypothesis of multiple equilibria and covers the drawbacks related to the endogeneity issue. Since the reaction of the US ESM may change in response to shocks under several regimes p&p-the NAST, $\mathrm{MSARH}$ is a proper technique for endogenously identifying the states during the utilized period (Shirazi 2022; Shirazi and Šimurina 2022; Shirazi et al. 2021; Geng et al. 2016).

Specifically, statistical significance of estimated coefficients (probability values) and the minimum value of “the Akaike Information Criterion” (AIC) are suggested to determine the number of states. Therefore, the $\mathrm{MRSM}$ is presented by Hamilton (1989):

8 $${\mathrm{Y}}_{\mathrm{t}}-{\upmu }_{{\mathrm{S}}_{\mathrm{t}}}= \sum\nolimits_{\mathrm{i}=1}^{\mathrm{m}}{\mathrm{\varphi }}_{\mathrm{i}}\left({\mathrm{Y}}_{\mathrm{t}-\mathrm{i}}- {\upmu }_{{\mathrm{S}}_{\mathrm{t}-\mathrm{i}}}\right)+ {\updelta }_{{\mathrm{S}}_{\mathrm{t}}}{\upvarepsilon }_{\mathrm{t}}$$

where ${\mathrm{Y}}_{\mathrm{t}}$ denotes the first difference of US' ESM, e.g.,${\mathrm{ESI}}_{\mathrm{I}}$,${\mathrm{ESI}}_{\mathrm{II}}$, ${\mathrm{ESI}}_{\mathrm{III}}$, and ${\mathrm{ESI}}_{\mathrm{IV}}$, $\upmu$ is the mean, and $\updelta$ is considered as the standard deviation of ${\mathrm{Y}}_{\mathrm{t}}$. As a discrete variable, ${\mathrm{S}}_{\mathrm{t}}$ $({\mathrm{S}}_{\mathrm{t}}\in \left\{1, 2, \dots ,\mathrm{ k}\right\})$ shows the first difference of the US ESM in different regimes. It is also noted that the standard deviation ($\updelta$) and mean ($\upmu$) of ${\mathrm{Y}}_{\mathrm{t}}$ are dependent on the specified regime ${\mathrm{S}}_{\mathrm{t}}$ for the time $\mathrm{t}$. Moreover, ${\mathrm{\varphi }}_{\mathrm{i}}$ is introduced as the parameters of the used model, and ${\upvarepsilon }_{\mathrm{t}}$ indicates a random variable with $\mathrm{i}.\mathrm{i}.\mathrm{d}\sim \mathrm{ N}(\mathrm{0,1})$.

Following Hamilton (1990), the state and discrete-time of the Markov switching process are applied for simulating ${\mathrm{S}}_{\mathrm{t}}$. Therefore, the transition matrix probabilities are indicated as:

9 $$\mathrm{P}= \left[\begin{array}{ccc}{\mathrm{P}}_{11}& \cdots & {\mathrm{P}}_{\mathrm{k}1}\\ \vdots & \ddots & \vdots \\ {\mathrm{P}}_{1\mathrm{k}}& \cdots & {\mathrm{P}}_{\mathrm{kk}}\end{array}\right]$$

where ${\mathrm{P}}_{\mathrm{ij}}=\mathrm{Pr}\left[{\mathrm{S}}_{\mathrm{t}}=\mathrm{j}\left|{\mathrm{S}}_{\mathrm{t}-1}=\mathrm{i}\right.\right]$ with ${\mathrm{P}}_{\mathrm{i}1}+{\mathrm{P}}_{\mathrm{i}2}+\dots +{\mathrm{P}}_{\mathrm{ik}}=1$ for all $\mathrm{i}$. Hamilton (1990) suggests the maximum-likelihood method to estimate the aforementioned parameters. Also, the value of ${\mathrm{S}}_{\mathrm{t}}$ equals $\mathrm{j}$ as ${\upvarepsilon }_{\mathrm{t}}$ is $\mathrm{i}.\mathrm{i}.\mathrm{d}\sim \mathrm{ N}(\mathrm{0,1})$ and hence, the conditional probability-density function of the variable ${\mathrm{Y}}_{\mathrm{t}}$ is:

10 $$\mathrm{f}({\mathrm{Y}}_{\mathrm{t}}\left|{\mathrm{S}}_{\mathrm{t}}=\mathrm{j}, {\mathrm{I}}_{\mathrm{t}-1};\uptheta )= \frac{1}{\sqrt{2\uppi {\upsigma }_{\mathrm{j}}}}\mathrm{exp}\left[-\frac{{({\mathrm{Y}}_{\mathrm{t}}-{\upmu }_{\mathrm{j}})}^{2}}{2{\upsigma }_{\mathrm{j}}^{2}}\right]\right.$$

where ${\mathrm{I}}_{\mathrm{t}-1}$ exhibits the captured information till $\mathrm{t}-1$. Accordingly, $\uptheta =({\upmu }_{1}, {\upmu }_{2},\dots , {\upmu }_{\mathrm{k}}; {\upsigma }_{1}, {\upsigma }_{2},\dots , {\upsigma }_{\mathrm{k}})$ presents the vector of parameters to estimate through the model. Furthermore, as ${\mathrm{I}}_{\mathrm{t}-1}$ is conditional, then the probability $\mathrm{f}({\mathrm{S}}_{\mathrm{t}}=\mathrm{j}\left|{\mathrm{I}}_{\mathrm{t}-1};\uptheta \right.)$ is known. Therefore, the probability density of the variable ${\mathrm{Y}}_{\mathrm{t}}$ is written as:

11 $$\mathrm{F}\left({\mathrm{S}}_{\mathrm{t}}=\mathrm{j}\left|{\mathrm{I}}_{\mathrm{t}-1};\uptheta \right.\right)=\mathrm{P}\left({\mathrm{S}}_{\mathrm{t}}=1\left|{\mathrm{I}}_{\mathrm{t}-1};\uptheta \right.\right)\mathrm{F}\left({\mathrm{Y}}_{\mathrm{t}}\left|{\mathrm{S}}_{\mathrm{t}}=1, \right. {\mathrm{I}}_{\mathrm{t}-1};\uptheta \right)+\mathrm{P}\left({\mathrm{S}}_{\mathrm{t}}=2\left|{\mathrm{I}}_{\mathrm{t}-1};\uptheta \right.\right)\mathrm{F}\left({\mathrm{Y}}_{\mathrm{t}}\left|{\mathrm{S}}_{\mathrm{t}}=2, \right. {\mathrm{I}}_{\mathrm{t}-1};\uptheta \right)+\dots +\mathrm{P}\left({\mathrm{S}}_{\mathrm{t}}=\mathrm{k}\left|{\mathrm{I}}_{\mathrm{t}-1};\uptheta \right.\right)\mathrm{F}\left({\mathrm{Y}}_{\mathrm{t}}\left|{\mathrm{S}}_{\mathrm{t}}=\mathrm{k}, \right. {\mathrm{I}}_{\mathrm{t}-1};\uptheta \right)$$

Moreover, the log-likelihood criteria for the observable time period is:

12 $$\mathrm{lnF}\left(\uptheta \right)=\frac{1}{\mathrm{n}}\sum\nolimits_{\mathrm{t}=1}^{\mathrm{n}}\mathrm{lnF}\left({\mathrm{Y}}_{\mathrm{t}}\left|{\mathrm{I}}_{\mathrm{t}-1}\right.;\uptheta \right)$$

Also, the maximum log-likelihood criteria is mentioned for the model coefficients to be estimated. Then, the state probability of ${\mathrm{S}}_{\mathrm{t}}$ is denoted as:

13 $$\mathrm{P}\left({\mathrm{S}}_{\mathrm{t}}=\mathrm{j}\left|{\mathrm{I}}_{\mathrm{t}};\uptheta \right.\right)=\frac{\mathrm{F}\left({\mathrm{S}}_{\mathrm{t}}=\mathrm{j}\left|{\mathrm{I}}_{\mathrm{t}-1};\uptheta \right.\right)\mathrm{F}\left({\mathrm{Y}}_{\mathrm{t}}\left|{\mathrm{S}}_{\mathrm{t}}=\mathrm{j}, \right. {\mathrm{I}}_{\mathrm{t}-1};\uptheta \right)}{\mathrm{F}\left({\mathrm{R}}_{\mathrm{t}}\left|{\mathrm{I}}_{\mathrm{t}};\uptheta \right.\right)}$$

The smooth probability considers that the probability of the different states is determined, applying the available information through the sample under consideration. Accordingly, the smoothed state probabilities are suggested for each regime at all the time points during the samples, based on Kim (1994). Hence, the smooth probabilities through the model are identified as follows:

14 $$\left({\mathrm{S}}_{\mathrm{t}}=\mathrm{j}\left|{\mathrm{I}}_{\mathrm{t}};\uptheta \right.\right)=\sum\nolimits_{\mathrm{i}=1}^{\mathrm{k}}\mathrm{P}\left({\mathrm{S}}_{\mathrm{t}}=\mathrm{j},{\mathrm{S}}_{\mathrm{t}+1}=\mathrm{i }\left|{\mathrm{I}}_{\mathrm{T}};\uptheta \right.\right)=\mathrm{P}\left({\mathrm{S}}_{\mathrm{t}}=\mathrm{j}\left|{\mathrm{I}}_{\mathrm{t}};\uptheta \right.\right).\sum\nolimits_{\mathrm{i}=1}^{\mathrm{k}}\frac{{\mathrm{P}}_{\mathrm{ji}}\times \mathrm{P}({\mathrm{S}}_{\mathrm{t}+1}=\mathrm{i }\left|{\mathrm{I}}_{\mathrm{T}};\uptheta )\right.}{\mathrm{P}({\mathrm{S}}_{\mathrm{t}+1}=\mathrm{i }\left|{\mathrm{I}}_{\mathrm{t}};\uptheta )\right.}$$

Finally, the expected time duration of specified regimes is determined from the transition probability of ${\mathrm{P}}_{\mathrm{jj}}$. Specifically, the expected duration of regime $\mathrm{j}$ is as follows:

15 $${\mathrm{D}}_{\mathrm{jj}}= \frac{1}{(1-{\mathrm{P}}_{\mathrm{jj}})}$$

Consequently, the behavioral properties of $\mathrm{DoPED}$ and $\mathrm{NCFP}$ indices of the US ES are affected through the NAST as follows:

if Uncertainty of “Upward” Regime (σ)$\downarrow \uparrow$ & Speed-and Expected Duration of “Upward” Regime $\uparrow \downarrow \stackrel{\mathrm{yields}}{\to }$ Risk $\downarrow \uparrow$ & Resilience $\uparrow \downarrow \stackrel{\mathrm{yields}}{\to }$ Energy Security $\uparrow \downarrow$

While the effect of the NAST on the behavioral characteristics of NEID and NCFP of the US ES is summarized as:

if Uncertainty of “Downward” Regime (σ)$\downarrow \uparrow$ & Speed- and Expected Duration of “Downward” Regime $\uparrow \downarrow \stackrel{\mathrm{yields}}{\to }$ Risk $\downarrow \uparrow$ & Resilience $\uparrow \downarrow \stackrel{\mathrm{yields}}{\to }$ Energy Security $\uparrow \downarrow$

## Results and discussion

### Results

#### Actual, long-term trends, and cyclical movements of the US ESM

The ES of an economy develops as the higher values of ES indices ${\mathrm{ESI}}_{\mathrm{I}}$ and ${\mathrm{ESI}}_{\mathrm{III}}$, and also fewer levels of ${\mathrm{ESI}}_{\mathrm{II}}$ and ${\mathrm{ESI}}_{\mathrm{IV}}$ are detected. However, the potential different reaction of the ESM in response to the NAST may be explained by the sensitivity level of energy sources (e.g., renewable and nonrenewable) consumption and net import for the specified indicators. Also, the different roles of crude oil and other suggested energy resources should be considered to analyze the mentioned reactions (Babajide 2017). Notably, the energy prices affect the diversification of primary energy supply that entails harnessing new energy resources, which is conducive to the resource equitability and abundance and switching to non-carbon-based fuel portfolio (Shirazi and Fuinhas 2023).The findings may lead to a structural framework that is supposed to enhance the US ES and promote sustainable economic development. In the following, the actual, long-term trends, and short-term fluctuations of the US' ESM are presented.

**Fig. 3:**
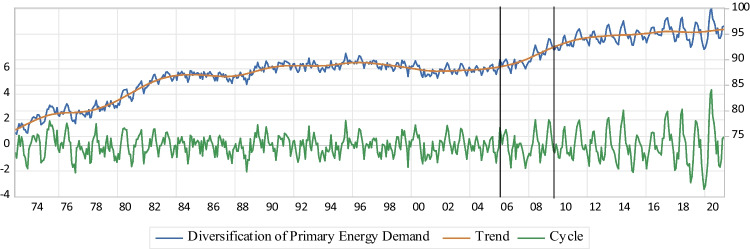
DoPED
- *NEID:* *ESI*_*II*_
  Following Fig. 4, $\mathrm{NEID}$ of the US economy (${\mathrm{ESI}}_{\mathrm{II}}$) has a slowly increasing trend (a relatively flat slop) before the NAST, while a moderate increase is experienced by ${\mathrm{ESI}}_{\mathrm{II}}$ after the NAST. Also, the minimum and maximum actual values of the US $\mathrm{NEID}$ are 98.8 and 99%, respectively which are high values before the NAST, while they are 98.99 and 99.3% after the NAST, showing no sign of a decreasing trend in response to the NAST. Furthermore, a fewer resilience regarding $\mathrm{NEID}$ is identified, since the magnitudes of ups and downs for short-term fluctuations of ${\mathrm{ESI}}_{\mathrm{II}}$ are considerably increased, after the NAST. Hence, the overall results exhibit that the US economy highly relies on energy resource imports p&p-the NAST. As a consequence, higher risk and less resilience are illustrated for the US ES, and therefore, there is a limited possibility to meet its energy consumption through domestic supply sources.

- *DoPED:* *ESI*_*I*_
  The Hodrick and Prescott (1997) decomposition of $\mathrm{DoPED}$ of the US economy (${\mathrm{ESI}}_{\mathrm{I}}$) shows an increasing trend with actual values from 75.4 to 91.2% pre-the NAST, with minimum 91.89% and maximum 99.9% values after the NAST. It indicates that the US economy’s energy supply sources have been getting more equally distributed among the major primary energy sources and therefore, a fewer risk of the US ES is concluded after the NAST. Moreover, the results exhibit that the NAST leads to greater magnitudes, and also fewer ups and downs for the short-term fluctuations of $\mathrm{DoPED}$, which is another implication of the US ES development in terms of higher resilience after the NAST (Fig. 3).

**Fig. 4:**
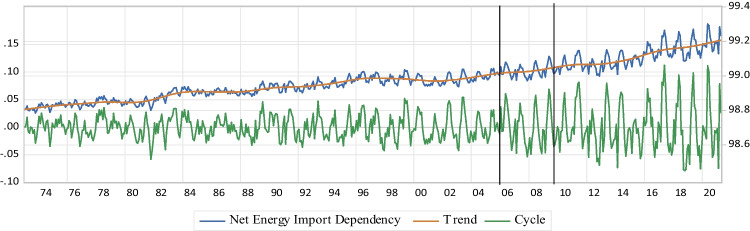
NEID
- *NCFP:* *ESI*_*III*_
  The third ES indicator is the $\mathrm{NCFP}$ of the US economy (${\mathrm{ESI}}_{\mathrm{III}}$) which shows a slowly increasing trend before the NAST, while a significant increase is detected for ${\mathrm{ESI}}_{\mathrm{III}}$ after the NAST (Fig. 5). Also, the actual values of the US $\mathrm{NCFP}$ are low and changing from 6.5 to 16.5% before the NAST, with the minimum 15.7% and maximum 24.9% values after the NAST. Furthermore, the results indicate no significant changes in the magnitudes and numbers of ups and downs for short-term fluctuations (resilience) of the US $\mathrm{NCFP}$ after the NAST. Therefore, and as the result of the NAST, a moderate potential offset to lower ${\mathrm{CO}}_{2}$-related environmental degradation of the US ES is concluded.

**Fig. 5:**
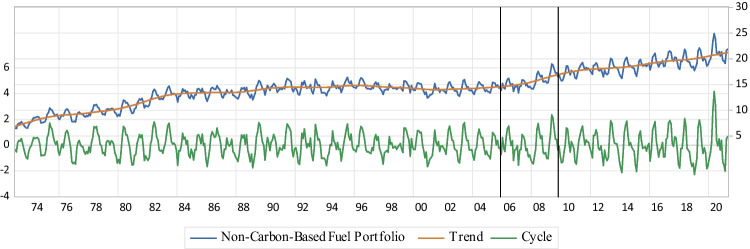
NCFP
- *NOID:* ESI_IV_
  The fourth ES indicator of the US economy (${\mathrm{ESI}}_{\mathrm{IV}}$) is $\mathrm{NOID}$ (Fig. 6). The actual time-series of ${\mathrm{ESI}}_{\mathrm{IV}}$ exhibit an increasing trend before the NAST, while a considerable decrease is detected for ${\mathrm{ESI}}_{\mathrm{IV}}$ after the NAST. Also, the minimum and maximum actual values of the US $\mathrm{NOID}$ are 4.5–24.5%, respectively pre-the NAST, while they decrease from 23.6 to 5.5% after the NAST. Furthermore, the results indicate moderate changes in the magnitudes and numbers of ups and downs for the short-term fluctuations of the US $\mathrm{NOID}$, after the NAST. Therefore, $\mathrm{NOID}$ of the US economy is negatively affected by the NAST, and a fewer risk with no considerable change in the resilience of the US ES is found as well, after the NAST.

**Fig. 6:**
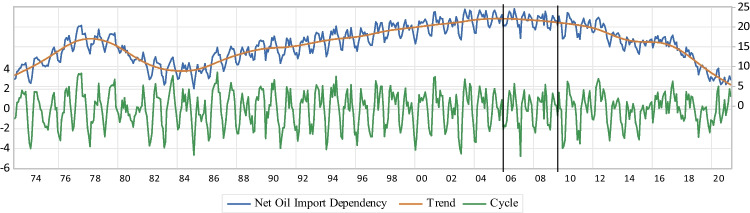
NOID

#### Descriptive statistics and unit root tests

In the next step, this study investigates the descriptive statistics and stationarity of the US ESM to support the pre-requisites of the $\mathrm{MRSM}$. Accordingly, and based on Table 1, all calculated time-series of the US ESM are recognized leptokurtic and skewed p&p-the NAST. Furthermore, they may demonstrate asymmetric or tail dependence behaviors and may have fully different types of marginal distributions.

**Table 1:** Descriptive statistics of the US ESM

Pre-the NAST								
Index	Mean	Median	Max	Min	Std.Dev	Skewness	Kurtosis	Jarque–Bera (Prob)
${\mathrm{ESI}}_{\mathrm{I}}$	85.91	87.36	91.24	75.44	3.8	− 1.09	2.9	79.5 (0.00)
${\mathrm{ESI}}_{\mathrm{II}}$	98.91	98.92	99.04	98.78	0.06	− 0.15	1.99	18.1 (0.00)
${\mathrm{ESI}}_{\mathrm{III}}$	12.76	13.65	16.41	6.45	2.37	− 0.9	2.73	55.6 (0.00)
${\mathrm{ESI}}_{\mathrm{IV}}$	15.42	15.34	24.48	4.48	4.5	− 0.1	2.19	11.1 (0.00)
Post-the NAST								
${\mathrm{ESI}}_{\mathrm{I}}$	94.83	94.74	99.99	91.89	1.62	0.49	3.1	5.8 (0.06)
${\mathrm{ESI}}_{\mathrm{II}}$	99.11	99.11	99.3	98.99	0.06	0.54	2.83	7.2 (0.03)
${\mathrm{ESI}}_{\mathrm{III}}$	18.95	18.75	24.9	15.7	1.62	0.66	3.92	15.1 (0.00)
${\mathrm{ESI}}_{\mathrm{IV}}$	15.4	16.2	23.6	5.5	4.7	-0.52	2.42	8.3 (0.02)

Then, the unit root tests based on automatic bandwidth selection of (Newey and West 1994; Andrews 1991) as well as breakpoint unit root test procedure support the conclusion that all the US ESM are stationary at the 1% statistical significance level in their first differences p&p- the NAST (Table 2). As a consequence, the $\mathrm{MRSM}$ is applicable to justify the behavioral states of the first difference of the US ESM (Bai and Lam 2019).

**Table 2:** Unit root test of the US ESM

Pre-the NAST	Level		First difference	
Unit root test	Adj. t-Stat (Prob)	Breakpoint (Prob)	Adj. t-Stat (Prob)	Breakpoint (Prob)
${\mathrm{ESI}}_{\mathrm{I}}$	− 3.3 (0.07)	− 4.1 (0.3)	− 29.3 (0.00)	− 23.7 ($<0.0$ 1)
${\mathrm{ESI}}_{\mathrm{II}}$	− 7.4 (0.00)	− 7.7 ($<0.0$ 1)	− 46.7 (0.00)	− 22.3 ($<0.01$)
${\mathrm{ESI}}_{\mathrm{III}}$	− 4.1 (0.01)	− 4.6 (0.1)	− 25 (0.00)	− 17.8 ($<0.01$)
${\mathrm{ESI}}_{\mathrm{IV}}$	− 5.1 (0.00)	− 5.4 ($<0.01$)	− 23.7 (0.00)	− 16.7 ($<0.01$)
${\mathrm{ESI}}_{\mathrm{I}}$	− 4 (0.01)	− 4.3 (0.2)	− 9.7 (0.00)	− 10.4 ($<0.0$ 1)
${\mathrm{ESI}}_{\mathrm{II}}$	− 4.8 (0.00)	− 5.8 ($<0.01$)	− 27.8 (0.00)	− 13.1 ($<0.01$)
${\mathrm{ESI}}_{\mathrm{III}}$	− 3.8 (0.03)	− 4.9 (0.03)	− 13.6 (0.00)	− 12.6 ($<0.01$)
${\mathrm{ESI}}_{\mathrm{IV}}$	− 3.6 (0.03)	− 5.03 ($0.03$)	− 20.9 (0.00)	− 11.6 ($<0.0$ 1)

#### Results of MSARH of the US ESM

The regimes of the US ESM are explained by the number of states, which are determined on the statistical significance of probability values related to the estimated coefficients and minimum of the AIC statistic. Accordingly, and based on the value and the sign of the estimated parameters, two “downward” (decrease state) and “upward” (increase state) regimes of the indices are classified in this paper (Shirazi and Šimurina 2022; Shirazi et al. 2021; Geng et al. 2016; Zhang and Zhang 2015; Artis et al. 2004; Ferrara 2003)^16^. Specifically, the “downward” regime (“upward” regime) is assessed as the sign of the estimated parameter is negative (positive) that shows the decrease (increase) state of the specified regimes.

- *a.   MSARH of the US'ESI*_*I*_

P&p-the NAST, the US $\mathrm{DoPED}$ shows two regimes. All parameter estimates of the regimes are found statistically significant (Table 3)^17^. The two regimes are summarized as “upward” and “downward”. As the regime switches from “upward” to “downward”, the uncertainty ($\upsigma$) faced by the US $\mathrm{DoPED}$ increases after the NAST, indicating that the variations of the US $\mathrm{DoPED}$ are vulnerable to disruption by affecting factors when heading on the decrease phase, while they almost are the same before the NAST. The speed (magnitude)- of the “upward” regime is greater than the “downward” regime p&p-the NAST. Moreover, the expected duration- of ${\mathrm{ESI}}_{\mathrm{I}}$ in the “downward” regime (14.2 months) is considerably higher than the “upward” regime (1.6 months) after the NAST, while the same speed is detected before the NAST with a fewer duration level for the “downward” regime (2.25 months). Furthermore, the transition probabilities show that the “downward” regime (93%) is more probable to persist than the “upward” regime (37%) after the NAST, which is consistent with the regime expected duration results. According to the expected durations, a “downward” (“upward”) regime is the dominant or typical state of the US $\mathrm{DoPED}$ p&p the NAST. Consequently, $\mathrm{DoPED}$ mitigates the volatility of fuel prices, contributes to the fuel price stability, and hence develops the US ES in terms of risk and resilience (Kosai and Unesaki 2020a, b; García Mazo et al. 2020; Liu et al. 2020a, b; Sun et al. 2020; Groissböck and Gusmão 2020; Francés et al. 2013; Roques et al. 2008)^18^. In respect of diagnostic tests, the findings of Durbin-Watson (DW) statistics pre- (2.03) and post- (2.07) the NAST show that no autocorrelation problem in the residuals is assessed in both sub-samples. Besides, the maximum Log-likelihood value (MLV) detects the goodness of fit through the models p&p the NAST.

**Table 3:** The $\mathrm{MSARH}$ of the US ${\mathrm{ESI}}_{\mathrm{I}}$

Pre-the NAST					
Dependent Variable:${\mathrm{ESI}}_{\mathrm{I}}$, AR (1)					
“Upward” Regime			“Downward” Regime		
Variables	Coefficients		Variables		Coefficients
C	0.56***		C		− 0.4***
$\upsigma$	0.49***		$\upsigma$		0.46 ***
Transition Probability					
Regimes		“Upward”		“Downward”	
“Upward”		0.47		0.53	
“Downward”		0.44		0.56	
Expected Duration					
“Upward” Regime: 1.88			“Downward” Regime: 2.25		
Durbin-Watson: 2.03			Log-likelihood: − 405.6		
Post-the NAST					
Dependent Variable: ${\mathrm{ESI}}_{\mathrm{I}}$, AR (6)					
“Upward” Regime			“Downward” Regime		
Variables	Coefficients		Variables		Coefficients
C	0.14***		C		− 0.06**
$\upsigma$	0.02***		$\upsigma$		0.9
Transition Probability					
Regimes		“Upward”		“Downward”	
“Upward”		0.37		0.63	
“Downward”		0.07		0.93	
Expected Duration					
“Upward” Regime: 1.6			“Downward” Regime: 14.2		
Durbin-Watson: 2.07			Log-likelihood: − 166.7

- *b.   MSARH* *of the US* *ESI*_*II*_

Based on Table 4, the US $\mathrm{NEID}$ shows “downward” and “upward” regimes p&p the NAST. As the regime switches, the uncertainty faced by ${\mathrm{ESI}}_{\mathrm{II}}$ is relatively stable p&p-the NAST, indicating that the short-term fluctuations of ${\mathrm{ESI}}_{\mathrm{II}}$ are invulnerable to disruption by affecting factors when the regimes change. Moreover, the speed or size- of the “upward” regime is greater than the “downward” regime, which exhibits that the US $\mathrm{NEID}$ has proceeded to indicate a fast upward- and sluggish downward movements post-the NAST, whereas the same speed is detected before the NAST. Furthermore, the NAST causes markedly higher speed for the “upward” regime, while the speed of the “downward” regime is not affected. Consistent with transition probabilities, the “downward” regime of ${\mathrm{ESI}}_{\mathrm{II}}$ has a larger expected duration (6.06 months) after the NAST. The “downward” regime is therefore the dominant regime of the US’ $\mathrm{NEID}$ post-the NAST, and also no dominant state is detected pre-the NAST. Consequently, in respect of risk and resilience, ES enhancement is achievable through the energy hubs, cross-border transactions in energy infrastructures and energy technologies, e.g., storage technologies and the shale development that is aligned with (Yong et al. 2021; Jiang et al. 2021; Coester et al. 2020; 2018; Rajavuori and Huhta 2020; Bekhrad et al. 2020; Azzuni and Breyer 2018)^19^. Notably, the findings of DW statistics pre- (1.97) and post- (1.77) NAST indicate no existence of autocorrelation in the models’ residuals during both sub-samples. Also, the MLV suggest the goodness of fit for the models’ p&p-the NAST.

**Table 4:** The $\mathrm{MSARH}$ of the US ${\mathrm{ESI}}_{\mathrm{II}}$

Pre-the NAST					
Dependent Variable:${\mathrm{ESI}}_{\mathrm{II}}$, AR (1)					
“Upward” Regime			“Downward” Regime		
Variables	Coefficients		Variables		Coefficients
C	0.01***		C		− 0.01***
$\upsigma$	0.01***		$\upsigma$		0.01***
Transition Probability					
Regimes		“Upward”		“Downward”	
“Upward”		0.36		0.64	
“Downward”		0.64		0.36	
Expected Duration					
“Upward” Regime: 1.57			“Downward” Regime: 1.56		
Durbin-Watson: 1.97			Log-likelihood: 1031.7		
Post-the NAST					
Dependent Variable: ${\mathrm{ESI}}_{\mathrm{II}}$, AR (4)					
“Upward” Regime			“Downward” Regime		
Variables	Coefficients		Variables		Coefficients
C	0.07***		C		− 0.01***
$\upsigma$	0.04***		$\upsigma$		0.02***
Transition Probability					
Regimes		“Upward”		“Downward”	
“Upward”		0.07		0.93	
“Downward”		0.16		0.84	
Expected Duration					
“Upward” Regime: 1.08			“Downward” Regime: 6.06		
Durbin-Watson: 1.77			Log-likelihood: 270.3

- *c.  MSARH* *of the US* *ESI*_*III*_

Before and after the NAST, the movements of the US $\mathrm{NCFP}$ have two significant security regimes (Table 5), which are summarized as “upward” and “downward”. However, the US $\mathrm{NCFP}$ presents the characteristics of decrease as well as increase p&p-the NAST. As the regime switches from “upward” to “downward”, the uncertainty faced by the ${\mathrm{ESI}}_{\mathrm{III}}$ decreases after the NAST, while they almost are the same before the NAST. The speed or size- of the “upward” is greater in comparison with the “downward” pre-the NAST, showing a slow decrease and steep increase in reaction to the NAST, while they are similar after the NAST. Consistent with state transition probabilities, the expected duration of ${\mathrm{ESI}}_{\mathrm{III}}$ in the “downward” regime (5.27 months) is higher than the “upward” regime (2.26 months) before the NAST, while the “downward”- and the “upward” regimes show the same expected duration (3.6 months) after the NAST. According to the expected duration of the movement regimes, the “upward” regime is the dominant state of the US $\mathrm{NCFP}$ pre-NAST, whereas no dominant state is detected post-NAST. It is concluded that focusing on $\mathrm{NCFP}$ decreases the costs of the US energy environment in terms of risk and resilience, and hence less ${\mathrm{CO}}_{2}$-related environmental degradation is assessed (Taherahmadi et al. 2021; Acemoglu et al. 2019; Gillessen et al. 2019; Anvar 2016; Jun et al. 2009; Lacasse and Plourde 1995)^20^. Moreover, the findings of the MLV and DW statistics exhibit no concern regarding the goodness of fit and autocorrelation in the residuals for both models.

**Table 5:** The $\mathrm{MSARH}$ of the US ${\mathrm{ESI}}_{\mathrm{III}}$

Pre-the NAST					
Dependent Variable:${\mathrm{ESI}}_{\mathrm{III}}$, AR (3)					
“Upward” Regime			“Downward” Regime		
Variables	Coefficients		Variables		Coefficients
C	0.62***		C		− 0.25***
$\upsigma$	0.43***		$\upsigma$		0.42***
Transition Probability					
Regimes		“Upward”		“Downward”	
“Upward”		0.58		0.42	
“Downward”		0.19		0.81	
Expected Duration					
“Upward” Regime: 2.36			“Downward” Regime: 5.27		
Durbin-Watson: 1.96			Log-likelihood: − 334.7		
Post-the NAST					
Dependent Variable: ${\mathrm{ESI}}_{\mathrm{III}}$, AR (3)					
“Upward” Regime			“Downward” Regime		
Variables	Coefficients		Variables		Coefficients
C	0.45**		C		− 0.42***
$\upsigma$	0.83***		$\upsigma$		0.46***
Transition Probability					
Regimes		“Upward”		“Downward”	
“Upward”		0.72		0.28	
“Downward”		0.28		0.72	
Expected Duration					
“Upward” Regime: 3.6			“Downward” Regime: 3.6		
Durbin-Watson: 1.91			Log-likelihood: − 166

- *d.   MSARH* *of the US* *ESI*_*IV*_

P&p-the NAST, the US $\mathrm{NOID}$ shows two states. All estimated parameters of both regimes are statistically significant (Table 6). The two states are called “downward” and “upward”. When the regime switches from “upward” to “downward”, the uncertainty faced by the US $\mathrm{NOID}$ markedly increases post-the NAST, indicating that movements of the US $\mathrm{NOID}$ are more vulnerable to disruption by affecting factors when the ${\mathrm{ESI}}_{\mathrm{IV}}$ faces the decrease phase, while it is stable pre-the NAST. The size- of the “downward” regime is the same as the “upward” regime post-the NAST, whereas it is relatively fewer than the “upward” regime before the NAST. Consistent with transition probabilities, after the NAST, the “downward” state of the US $\mathrm{NOID}$ has the larger expected duration (24.4 months), while the two regimes are relatively the same in expected duration before the NAST. The “downward” state is therefore the dominant state of ${\mathrm{ESI}}_{\mathrm{IV}}$ after the NAST. Accordingly, focusing on the advantages of NAST declines $\mathrm{NOID}$ of the US economy, and hence, the reliable energy supplies can mitigate volatility and vulnerability of the energy system, in the respect of risk and resilience (Acemoglu et al. 2019; Pérez et al. 2019; Novikau 2019; Gillessen et al. 2019; Zaman and Brudermann 2018)^21^. Besides, the findings of the MLV and the DW statistics indicate that neither “misspecification of the functional form” nor “autocorrelation in residual terms” is not an issue through the models.

**Table 6:** The $\mathrm{MSARH}$ of the US ${\mathrm{ESI}}_{\mathrm{IV}}$

Pre-the NAST					
Dependent Variable:${\mathrm{ESI}}_{\mathrm{IV}}$, AR (3)					
“Upward” Regime			“Downward” Regime		
Variables	Coefficients		Variables		Coefficients
C	0.79***		C		− 0.62**
$\upsigma$	1.05		$\upsigma$		1.1
Transition Probability					
Regimes		“Upward”		“Downward”	
“Upward”		0.29		0.71	
“Downward”		0.61		0.39	
Expected Duration					
“Upward” Regime: 1.4			“Downward” Regime: 1.63		
Durbin-Watson: 2.06			Log-likelihood: − 666		
Post-the NAST					
Dependent Variable: ${\mathrm{ESI}}_{\mathrm{IV}}$, AR (3)					
“Upward” Regime			“Downward” Regime		
Variables	Coefficients		Variables		Coefficients
C	0.1***		C		− 0.09***
$\upsigma$	0.004***		$\upsigma$		1.17**
Transition Probability					
Regimes		“Upward”		“Downward”	
“Upward”		0.02		0.98	
“Downward”		0.04		0.96	
Expected Duration					
“Upward” Regime: 1.01			“Downward” Regime: 24.4		
Durbin-Watson: 2.07			Log-likelihood: − 209.6		

### Discussion

Based on the calculated values of the first ES index, ${\mathrm{ESI}}_{\mathrm{I}}$, the equitability dimension of the US' $\mathrm{DoPED}$ are increased gradually pre-the NAST, while a significant take-off with more persistent ups and downs is detected after the NAST (Fig. 3). From the aspect of uncertainty, the country exhibits less biodiversity in primary energy sources after the NAST, when ${\mathrm{ESI}}_{\mathrm{I}}$ faces the “downward” regimes. Also, and due to the higher speed of the “upward” regimes, the economy’s energy supply sources keep on taking the line of more equal distribution among the major primary energy sources post-NAST. Despite the overall positive impacts of the NAST on ${\mathrm{ESI}}_{\mathrm{I}}$, the dominant “downward” regime in p&p-the NAST is the sign of concerns for the biodiversity of the US primary energy sources (Table 3). Consequently, the interconnection of uncertainty, speed- and expected duration of specified switching regimes of $\mathrm{DoPED}$ lead to a combination of fewer risks and higher resilience of the US ES, in response to the NAST. Specifically, the NAST causes resource availability, and a negative correlation among energy prices that facilitates the replacement of coal, oil, and green energy sources (e.g., nuclear power and renewable energy resources) by natural gas in the energy mix that supports the physical availability, price affordability and accessibility dimensions of the US ES, and therefore, increases the US $\mathrm{DoPED}$ (Kosai and Unesaki 2020a, b; García Mazo et al. 2020; Hasanov et al. 2020; Liu et al. 2020a, b; Sun et al. 2020; Acemoglu et al. 2019; Francés et al. 2013).

Following the results of $\mathrm{NEID}$, the calculated values of ${\mathrm{ESI}}_{\mathrm{II}}$ are not decreased after the NAST that shows no sign of a considerable proper reaction to the NAST. Also, the short-term fluctuations of ${\mathrm{ESI}}_{\mathrm{II}}$ are significantly intensified after the NAST. This is the other sign of no ES development in response to the NAST in respect of $\mathrm{DoPED}$, and imports through the US energy systems (Fig. 4). From the aspect of uncertainty, the country becomes less vulnerable to disruption by affecting factors after the NAST, when ${\mathrm{ESI}}_{\mathrm{II}}$ faces the “downward” regimes. Also, and due to higher magnitudes of the “upward” regimes, the US economy gets more dependent on foreign primary energy sources to cover its $\mathrm{PED}$ post-the NAST. Despite the overall improper impacts of the NAST on ESI_II_, the dominant “downward” state after the NAST may lessen the concerns of the US dependency on domestic primary energy sources (Table 4). Hence, a mixture of higher risk and less resilience of ES is concluded for the US energy systems through the NAST, as the country has been getting highly relies on energy imports and therefore, there is a limited possibility to meet its energy consumption via domestic energy sources. Consequently, the US diversification and imports of energy sources should be re-designed (Gong et al. 2021; Lin and Raza 2020; Li et al. 2020; Augutis et al. 2020; Kosai and Unesaki 2020a, b; Gan et al. 2019).

Also, the increasing trend and relative stability of the cyclical movements of the third ES measurement (${\mathrm{ESI}}_{\mathrm{III}}$) indicate that the US economy is significantly successful to switch from $\mathrm{CFP}$ to $\mathrm{NCFP}$ after the NAST (Fig. 5). From the aspect of uncertainty, the vulnerability of the US economy to disruption by affecting factors is increased after the NAST, when ${\mathrm{ESI}}_{\mathrm{III}}$ enters the “upward” regimes. Also, the speed of “upward” regimes decreases after the NAST, leading to a higher risk with no markedly change in the resilience of potential offset in order to lower the US ${\mathrm{CO}}_{2}$-related environmental degradation, which can be intensified by no dominant “upward” regime post-the NAST (Table 5). Accordingly, the findings imply that the NAST improves the contribution level of hydro, nuclear, and $\mathrm{NRE}$ to total $\mathrm{PED}$ in the US primary energy systems, and hence a considerable decline in the US ${\mathrm{CO}}_{2}$-related environmental degradation is concluded. So, the intermediate technology of the NAST develops the ${\mathrm{CO}}_{2}$-related environmental, political, and social acceptability dimensions of the US ES since the price reduction of natural gas leads to the ${\mathrm{CO}}_{2}$ emission decline (Shirazi and Šimurina 2022; Sutrisno et al. 2021; Acemoglu et al. 2019; Gillessen et al. 2019; APERC 2007).

Finally, the actual time-series of ${\mathrm{ESI}}_{\mathrm{IV}}$ show a considerable change from an increasing to a decreasing trend after the NAST that exposes the share of the US economy's net oil imports in its total $\mathrm{PED}$ is decreased. Also, the short-term fluctuations of ${\mathrm{ESI}}_{\mathrm{IV}}$ get limited significantly after the NAST (Fig. 6). In contrast, the US concerns in respect of ES increase, when the “downward” regime of ${\mathrm{ESI}}_{\mathrm{IV}}$ takes place. Despite the speed of the “upward” and “downward” regimes decline after the NAST, the existence of a typical “downward” state of ${\mathrm{ESI}}_{\mathrm{IV}}$ may cause a fewer risk but no significant change in resilience for the US ES, after the NAST (Table 6). Therefore, the overall signals exhibit that $\mathrm{NOID}$ of the US economy is properly affected by the NAST in respect of sustainable development. Hence, and consistent with (Gan et al. 2019; Biresselioglu et al. 2015; 2012; Lacasse and Plourde 1995), the decreasing reliance on import energy resources reduces the country’s sensitivity to the effects of the external shocks occurred in the US energy-importing process. Notably, the comparison between the results of the second (${\mathrm{ESI}}_{\mathrm{II}}$) and fourth (${\mathrm{ESI}}_{\mathrm{IV}}$) US ESM reveals the successful outcome of the US economy in $\mathrm{NOID}$ after the NAST, while the country has not achieved any developments in import independence for the rest of primary energy resources^22^.

Therefore, the US ES can be enhanced in respect of “the energy trilemma” via the efficient interaction among the short-term effects, e.g., substitution and scale effect, and the long-term impacts of the NAST through $\mathrm{DoPED}$, $\mathrm{NCFP}$, and $\mathrm{NOID}$, while $\mathrm{NEID}$ declines the US ES, in terms of risk and resilience^23^. Accordingly, focusing on the energy-related concept of economic complexity, i.e., the strategic management, control and storage of energy supply, higher reserves of energy sources, optimized structure of the terminal sectors’ energy consumption, clean energy development, and energy efficiency improvement, which is the outcome of the NAST, is necessary to enhance 4 As dimensions of ES when internal and external shocks occur in the US primary energy systems.

Different priorities regarding diverse interpretations of ES require specialization in energy policies since countries with technically recoverable shale reserves are different in the institutional features, especially regarding the large-scale exploitation process of the shale reserves. Nevertheless, it is expected that switching from underdeveloped and developing technologies (scale effect) to the change in the institutional characteristics and intermediate technology (composition effect) and developed technologies (technique effect) lead to movement towards efficient shale industrialization process. Based on the results of this work and in respect of other countries' exploration to the shale reserves, utilization of the economies of scale in the shale technology develops the coordinating mechanism in the countries' energy systems. This process probably enables these countries to exploit their shale reserves commercially, which leads to signify utilization of the desirable explicit and implicit ES outcomes, especially for China, Argentina, Algeria, Mexico, Canada, Australia, Russia, South Africa, and Brazil that have technically recoverable shale reserves, but have not markedly started to extract the shale reserves due to the institutional and technological constraints.

However, the innovation and technology advancements of the shale reserves significantly escalate the shale oil and gas production, which cause undesirable market effects, and socio-environmental concerns, e.g., the methane emissions known as the by-product of the shale reserves, and marine pollution caused by large water intensity of the hydraulic fracturing (Bilgili et al. 2016; Wang et al. 2014), habitat destruction, and local anomalies (Mason et al. 2015) that are mentioned as the major limitations of this research and therefore, suggested to study by further investigations.

## Conclusions

While ES has explicit and implicit impacts on the economy, the US $\mathrm{PDPES}$ in terms of “the energy trilemma” shape geopolitics and affect global ES. The effect of the NAST on ES performance is known as a necessary condition to overcome the barriers on the way of vulnerability reduction and promotion of sustainable economic development for the USA as the world’s largest energy-consuming economy. Therefore, a comprehensive analysis is aimed in this research to examine time-varying and asymmetric behavioral characteristics of the US ES p&p-the NAST. The US ES is analyzed in this paper through the 4 As of primary energy resources using the $\mathrm{MSARH}$. To this end, four indices, e.g., $\mathrm{DoPED}$, NEID, NCFP, and NCFP are calculated to expose the importance and potential risks and benefits, regarding the US' $\mathrm{PDPES}$ p&p-the NAST. The findings indicate that the interconnection of uncertainty, speed and expected duration of the specified switching regimes of the measurements support the time-varying and asymmetric behavioral regimes for the US ESM p&p-the NAST. Also, the overall interaction of substitution effect and scale effect of the NAST develops the US ES in terms of risk and resilience, through $\mathrm{PDPES}$. Consequently, the relative policy implications are presented as follows:

- Facilitating $\mathrm{DoPED}$ via the offshore shale institutional improvements to mitigate the volatility of fuel prices and hence, contribute to the fuel price stability and long-term sustainability transitions.
- Developing the shale innovative and intermediate technologies, and the country’s commitments to NCFP via R&D loan guarantees to decrease the costs of the US energy environment and therefore, capture less ${\mathrm{CO}}_{2}$-related environmental degradation
- Alternating analysis of the risks and benefits of the shale and renewable energy technological changes to decline the $\mathrm{NEID}$ of the US economy and thus, more reliable energy supplies to meet its energy consumption through domestic supply sources
- Promoting resilience of the US energy systems through the strategic management, control and storage of energy supply, higher reserves of energy sources, clean energy development, optimization of the structure of terminal energy consumption, and energy efficiency improvement
- Adopting energy transport and trading improvement policies, regarding the accessibility along major resource trade routes

## Data Availability

Data is available upon request.

## Footnotes

1 See Cherp and Jewell (2014) for more details.

2 Geopolitical interests and events in the carbon-based energy market changes, which makes new and renewable energy portfolio appear more critical in the global energy security (Flouros et al. 2022; Øverland et al., 2017).

3 Different priorities regarding diverse interpretations of ES require specialization in energy policies (APERC 2007).

4 The affecting factors of energy system resilience refer to “technologic diversification”, “spare production capacities”, “diverse suppliers stockpiling”, and “emergency plans” (Yergin 2006).

5 The ES risk index assesses the annual countries' energy-related vulnerability in the world energy market, which applies quantifiable information, historical trend data, and governments' projections to recognize the policy decisions and other affecting factors that relate negatively or positively to the counties' ES (Global Energy Institute. The US Chamber of Commerce 2020).

6 See APERC (2007) for the details of the US' ES comprehensive roadmap, e.g., “Energy Policy Act 2005” and “Asia Pacific Partnership”.

7 Based on Bilgili et al. (2016), China, Argentina, Algeria, the US^A^, Canada, Mexico, Australia, South Africa, Russia, and Brazil are the countries with the largest technically recoverable shale reserves, respectively. However, these countries can't utilize shale gas as much as the US utilizes (Auping et al. 2016), which is due to the differences in the US and the countries with technically recoverable shale reserves, i.e., the institutional features and the large-scale exploitation process of the shale reserves (Tian et al. 2014; Kuuskraa et al. 2013).

8 See Mason et al. (2015) for more details in respect of benefits of the NAST for the US economy.

9 See ^the “^^Material^^”^ section of this article for more details.

10 Following APERC (2007), only the actual time-series of these four ESM are calculated and statically analyzed, for the Asia–Pacific Economic Cooperation (APEC) members during the time period before the NAST, which neither the impacts of the NAST on the behavior of actual time-series and its decompositions of ESM nor the rest of the aforementioned contributions of this article (especially the switching regimes) are addressed.

11 This approach is applicable to develop ES of the countries that have considerable technically recoverable shale reserves but haven't significantly started yet to extract the reserves due to the technological constraints, and also the economies that are net primary energy importers to cover their energy consumption.

12 It is worth noting that the comparison between the results of NEID and NCFP indices reveal the successful outcome of the US economy in net oil import independence after the NAST, while the country has not achieved any developments in import independence for the rest of primary energy resources.

13 The new and renewable energy sources are recognized locally scaled affordable, which have currently considered weak potential substitutes for the conventional energy sources, despite their importance to mitigate energy supply security concerns is growing. This is due to that the future physical limitations met by the accessibility dimension of ES are suggested to be reduced through the associated technology developments (APERC 2007).

14 - The two sub-periods to recognize p&p-the NAST are distinguished through the black vertical lines provided in Figs. 1^, 2, 3, 4, 5^, 6.

15 Based on the EIA data category, primary energy consumption by the source is classified as biomass, coal, natural gas, petroleum, nuclear, and total renewable, while primary energy net imports by source are sorted as biomass, coal, electricity, natural gas, crude oil, and petroleum. Accordingly, the primary energies listed for the consumption (caption, Fig. 1) and those for the import (caption, Fig. 2) are inconsistent.

16 Refereeing to (Zhang and Zhang 2015; Artis et al. 2004; Ferrara 2003), as the results show more than one “upward" or “downward'' regimes, the “slightly”, “moderately” and “sharply” regimes are detected based on the descending to ascending sizes or magnitudes of the estimated parameters, respectively.

17 Note: ***, **, * indicate 0.01, 0.05, 0.1 significant level, respectively.

18 Among member countries of Asia–Pacific Economic Cooperation (APEC), Canada, New Zealand, and Chile are projected to reduce the diversification of primary energy demand (APERC 2007).

19 As the diversification of primary energy demand decreases due to lack of domestic energy resources, the level of NEID increases, which is experienced by Japan, South Korea, Singapore, Chinese Taipei, Hong Kong, and Chile (APERC 2007).

20 Most countries around the world have not been successful in considerable environmental degradation through NCFP, showing the growth rate of non-carbon-based energy sources is not high enough to cover their future consumption growth (APERC 2007).

21 It is projected that among 21 member countries of APEC, 9 economies including the USA, China, Australia, Peru, Chile, Malaysia, Indonesia, Vietnam, and Thailand will develop their levels of ES in the terms of NCFP by 2030 (APERC 2007).

22 Based on Sutrisno et al. (2021), Acemoglu et al. (2019), Gan et al. (2019), Gillessen et al. (2019), Biresselioglu et al. (2015; 2012), APERC (2007), Lacasse and Plourde (1995), it is suggested that the oil supply security is significantly dependent on the movements in the contributions of energy consumption across oil intensive sectors, economic development, diversification of primary energy demand, and diversification of energy import supply.

23 The overall results present the asymmetric and time-varying behavioral regimes for the US ESM, e.g., diversification of primary energy demand, NEID, NCFP, and NCFP, pre-and post-the NAST.
